# Detection of a fish bone in the cervical esophagus using point-of-care ultrasound

**DOI:** 10.1186/2036-7902-7-S1-A22

**Published:** 2015-03-09

**Authors:** T Kameda

**Affiliations:** Department of Emergency Medicine, Red Cross Society Azumino Hospital, Azumino, Japan

**Keywords:** Public Health, Compute Tomography, Informed Consent, Emergency Department, Emergency Medicine

## Background

To our knowledge, the use of point-of-care ultrasound for detecting a fish bone in the cervical esophagus has never previously been reported in the English literature.

## Case presentation

We herein report the case of the accidental ingestion of a fish bone which was detected in the cervical esophagus by means of point-of-care ultrasound. A-70-year-old woman presented at the emergency department of our hospital with odynophagia in the neck after eating a slice of grunt (a type of fish) simmered in broth. Prior to this presentation she had already undergone direct laryngoscopy without any fish bone being identified at an otorhinolaryngology clinic. Point-of-care ultrasound was thereafter performed. As a result, a linear and hyperechoic structure was thus identified in the cervical esophagus. The structure did not seem to penetrate the wall of the esophagus. Computed tomography confirmed the same finding without any penetration. An endoscopic examination revealed a fish bone stuck in the cervical esophagus. The bone measured 17 mm in length, and was safely removed under endoscopic guidance. Subsequently the patient demonstrated a good recovery without any complications.

## Conclusion

We describe the identification of a fish bone in the cervical esophagus using point-of-care ultrasound. Point-of-care ultrasound may therefore be a useful modality for both detecting the presence of fish bones in the cervical esophagus and also for confirming the existence of any complications.

## Informed consent

The study was conducted in accordance with the ethical standards dictated by applicable law. Informed consent was obtained from each owner to enrolment in the study and to the inclusion in this article of information that could potentially lead to their identification.

